# Research on the Evolution Model of Grain Boundary Helium Bubbles in the Weld Heat-Affected Zone of Austenitic Stainless Steel After Irradiation

**DOI:** 10.3390/ma19214716

**Published:** 2026-11-05

**Authors:** Shaohua Yin, Zhiqiang Sun, Yingjie Chen, Zhaosheng Guo, Zhen Luo

**Affiliations:** 1 School of Materials Science and Engineering, Tianjin University, Tianjin 300350, China; jnyinshaohua@126.com; 2 Suzhou Nuclear Power Research Institute Co., Ltd., Suzhou 215004, China; 3 China Nuclear Power Operations Co., Ltd., Shenzhen 518124, China

**Keywords:** helium embrittlement, grain boundary bubbles, irradiated steel welding, multistage modeling, nuclear materials

## Abstract

The weldability of neutron-irradiated austenitic stainless steels in pressurized water reactors (PWRs) deteriorates significantly due to helium accumulation at grain boundaries (GB) originating from nuclear transmutation reactions. This study develops a multistage model to elucidate helium bubble evolution during welding, including nucleation, coalescence, high-temperature growth, and cooling-induced tensile stages, by quantitatively correlating bubble density/diameter with initial He concentration (0.2–20 appm (atomic parts per million)), peak temperature (1450 K, 1566 K, 1641 K, 1657 K), and stress (0–200 MPa). Key results demonstrate that He concentration nonlinearly affects bubble growth (threefold diameter increase at 20 appm versus 0.2 appm during cooling), while temperature governs bubble kinetics with critical threshold behavior (>900 K) and tensile stress promotes cooling-stage expansion over the investigated stress range. Excessive welding heat input prematurely terminates coalescence, increasing final bubble density/diameter and promoting He-induced cracking, consistent with the reported morphology and order of magnitude of grain-boundary cavities. The model provides a theoretical framework for predicting He embrittlement and optimizing stress-modified welding techniques (e.g., auxiliary beam laser welding) for nuclear component repair.

## 1. Introduction

**Figure 1:**
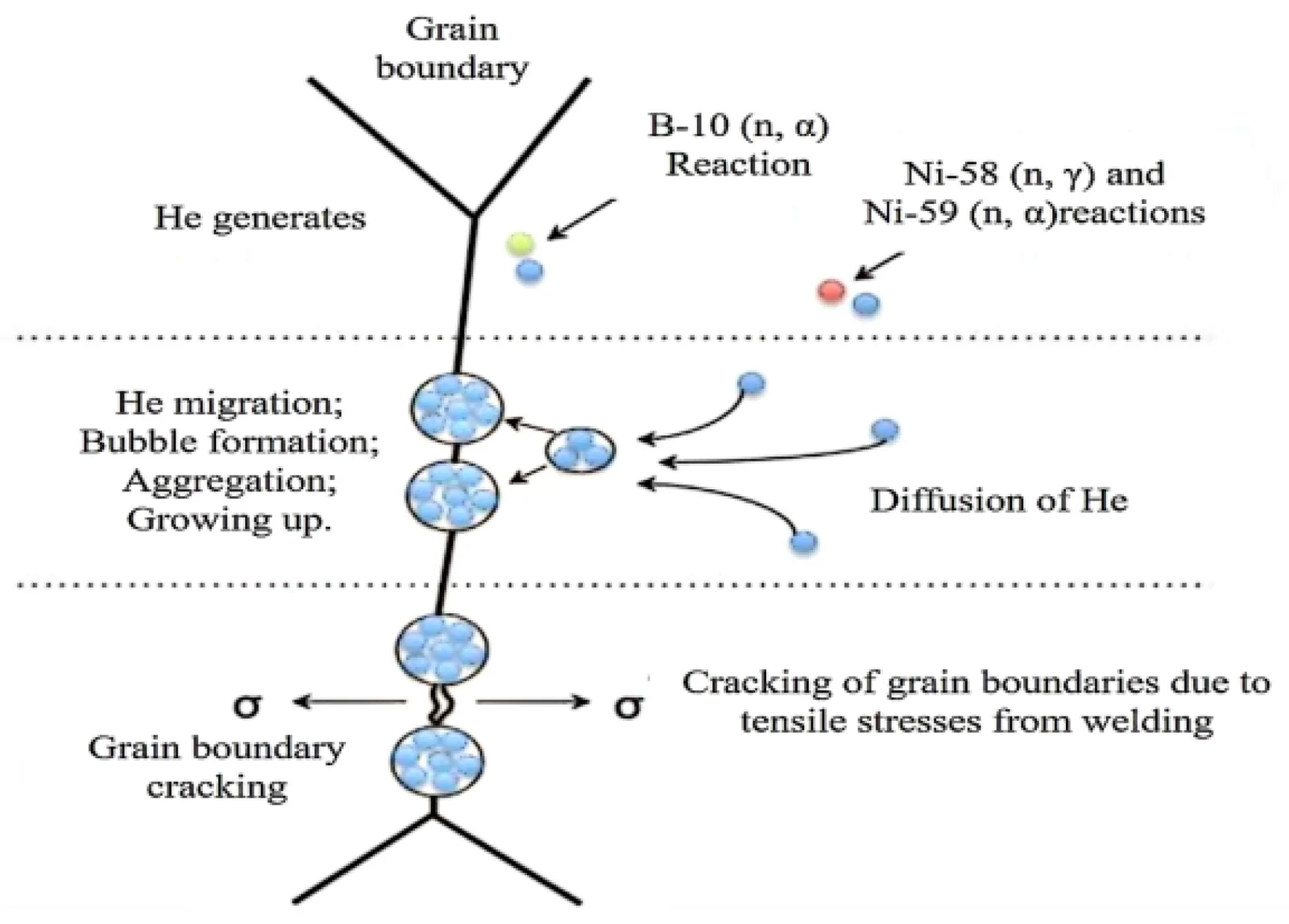
Schematic diagram of the formation mechanism of helium-induced cracks [5].

The existing pressurized water reactor nuclear power plants are gradually aging, and the weldability of austenitic stainless steel used in reactor pressure vessels and reactor core components is decreasing day by day. The main reason for the decline in weldability is that boron (B) and nickel (Ni) in austenitic stainless steel undergo nuclear transmutation reactions with neutrons to generate helium (He) [1,2,3]. The relevant nuclear transmutation reaction equations are ^10^B + (n, α)→^7^Li + ^4^He and ^58^Ni + (n, γ) → ^59^Ni + (n, α) → ^56^Fe + ^4^He. Since helium is essentially insoluble in the metal, the elevated welding temperature promotes its diffusion and trapping at irradiation-induced defects and pre-existing grain-boundary defects, which can subsequently serve as bubble nucleation sites. These trapping sites are not assumed to be pores, micropores, or fully developed helium bubbles. In addition, the presence of tensile stress at high temperature will accelerate the aggregation and growth of bubbles at the grain boundaries [4]. As the size and concentration of the bubbles increase, the grain boundaries gradually weaken. The tensile stress generated during the post-welding cooling acts on the grain boundaries. When the helium bubble concentration reaches the threshold concentration for crack generation, cracks will occur in the welding heat-affected zone. This is the microscopic mechanism for the generation of helium-induced cracks (helium induced crack, HeIC), and its cracking mechanism is shown in Figure 1 [5].

The evolution of helium bubbles at the grain boundaries during the welding process can be divided into four characteristic stages: nucleation, aggregation, high-temperature growth, and cooling-induced stretching. During the nucleation and aggregation stages, the grain boundaries have a significant influence on the evolution of helium bubbles; while during the high-temperature growth and cooling-induced stretching stages, the thermal stress generated by the welding heat input becomes the dominant factor in the evolution of helium bubbles.

The grain boundary, as a special region in the crystal structure, has high energy and disordered atomic arrangement, creating favorable conditions for the nucleation and aggregation of helium bubbles. The large-scale aggregation of helium bubbles at the grain boundaries will cause significant changes in the microstructure of the material, leading to the weakening of the grain boundaries and further deteriorating the mechanical properties of the material, such as a decrease in strength, a reduction in toughness, an increase in brittleness, etc., which seriously threatens the safe operation of nuclear facilities. For example, in a high-temperature environment, the helium bubbles at the grain boundaries may cause intergranular embrittlement of the material, resulting in premature failure of the material. Apart from the high-temperature degradation behavior of austenitic stainless steels under irradiation and welding thermal cycles, the material also exhibits distinctive microstructure evolution and mechanical responses under cryogenic extreme temperature conditions. A recent study, Serration-induced plasticity in phase transformative stainless steel 316L upon ultracold deformation at 4.2 K [6], reported the tensile deformation behavior and microstructural evolution of 316L stainless steel at liquid helium temperature (4.2 K). This work revealed that the ultracold loading condition can trigger coupled phase transformation and twinning mechanisms, accompanied by serrated plastic flow, which leads to simultaneous improvement in strength and ductility. This finding demonstrates that austenitic stainless steels show highly temperature-dependent deformation mechanisms across a wide temperature range from liquid helium temperature up to the high-temperature welding regime. Therefore, it is essential to understand the microstructure evolution of austenitic stainless steel under extreme temperature conditions for nuclear component service and repair.

M.N. Gussev et al. [7] used scanning electron microscopy, electron backscatter diffraction, and transmission electron microscopy (TEM) methods to study the microstructure of the weld seam and the heat-affected zone (HAZ). They observed signs of early damage related to helium in the heat-affected zone, which were manifested as degraded grain boundaries (GB) “decorated” by chains of pores and most fine (i.e., ≤25–30 μm) dispersed cracks. The crystallographic analysis of the HAZ damage showed that random high-angle grain boundaries are most susceptible to damage related to helium. Cheik Njifon [8] studied the behavior and influence of helium in alloy 800H and found that there is a significant helium accumulation phenomenon at the grain boundaries, while the adjacent areas are helium-depleted regions. The combination of the defect structure and the high density of helium clusters at the grain boundaries leads to a significant degradation of the mechanical properties of the single crystal and bicrystal of alloy 800H.

**Figure 2:**
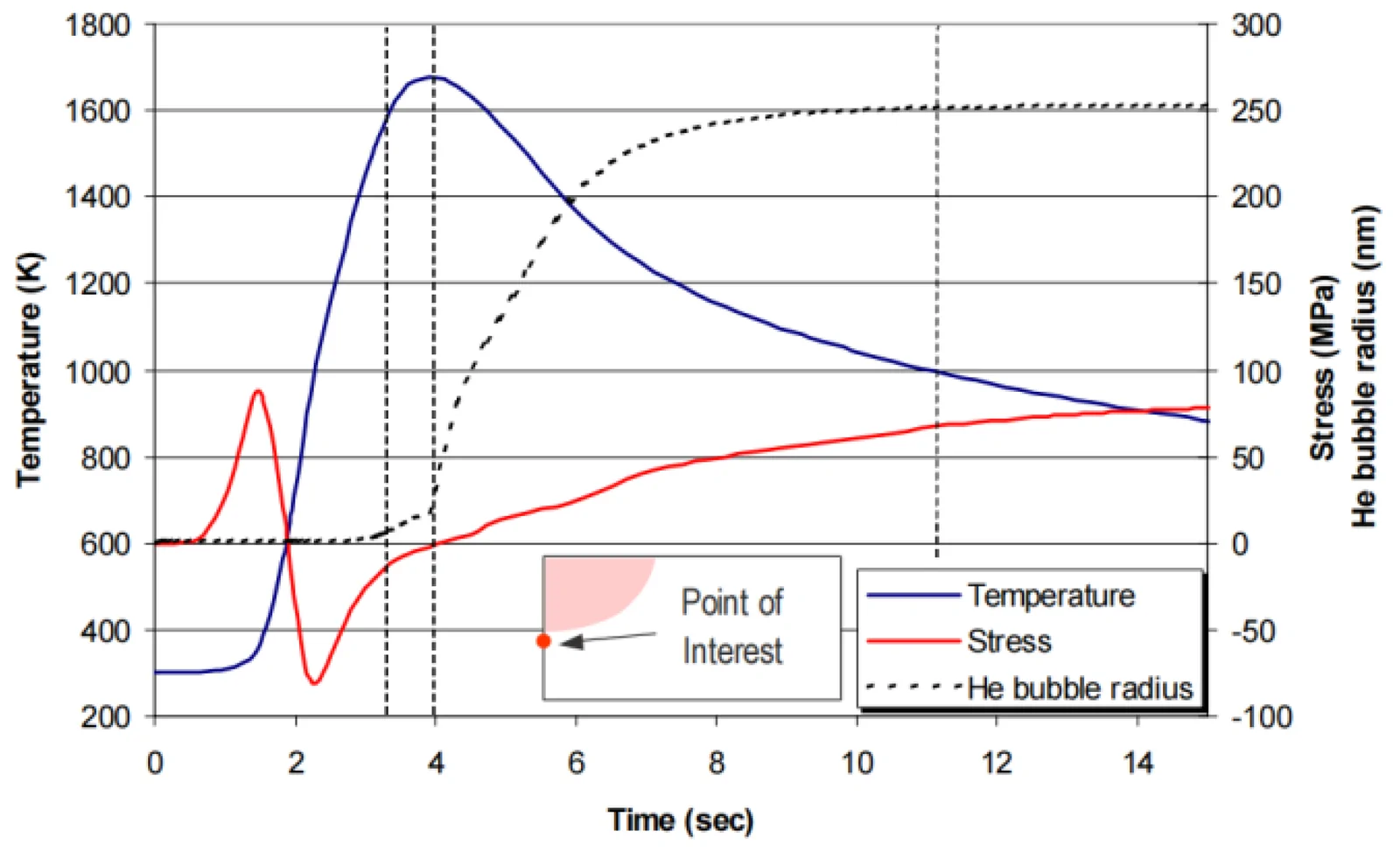
Relationship among temperature, stress, and helium bubble growth [7].

The influence of the stress state on HeIC has been fully confirmed [7,8,9,10]. The growth of helium bubbles depends on the presence and magnitude of the tensile stress. Z. Feng et al. [10] obtained the relationship between the welding thermal cycle, stress state, and the growth rate of helium bubbles through a modeling method, as shown in Figure 2. Figure 2 shows the changes in temperature, average equivalent stress, and the radius of helium bubbles over time at a point of interest about 0.5 mm outside the fusion line. This point is located below the centerline weld seam, with a peak temperature of 1644 K and a welding heat input of 7.1 kJ/cm, which is consistent with the experiment of Asano et al. As can be seen from Figure 2, when the arc passes through the point of interest, the temperature, stress, and bubble growth will all change dynamically. During the heating stage below 600 K, although there is a certain level of tensile stress, the size of the He bubbles is zero; during the heating stage from 600 K to about 1600 K, the size of the helium bubbles is still zero under the action of compressive stress; during the heating stage from 1600 K to the highest temperature, the size of the helium bubbles increases slightly (the first stage of growth) under the action of lower compressive stress, and at this time, the radius of the helium bubbles reaches 17 nm; when cooling from the highest temperature to 1000 K, as the tensile stress increases, the size of the He bubbles increases sharply and reaches a peak of 252 nm at 1000 K (the second stage of growth). When the temperature drops below 1000 K, even if the stress continues to increase, the growth of the bubbles becomes negligible.

Lin, H.T. et al. [3]. believe that the growth of helium bubbles in the first stage is caused by the excessive helium pressure inside the bubbles due to high temperature. The growth of helium bubbles in the second stage is due to the absorption of vacancies induced by tensile stress. The simulation results of Z. Feng et al. show that the growth of helium bubbles induced by stress is much faster than that driven by high temperature.

In this study, an evolution model of grain boundary helium bubbles based on the above four characteristic stages was constructed, fully considering key factors such as the initial concentration of helium, temperature, and stress. The model uses the density and diameter of helium bubbles as characterization parameters to systematically describe the evolution process of helium bubbles at each stage. For different characteristic stages, the model respectively established control equations describing the main factors affecting the growth of bubbles, fully demonstrating the stage characteristics of bubble evolution. The model divides the evolution process of helium bubbles at the grain boundaries during the welding process into four stages: nucleation, aggregation, high-temperature growth, and cooling-induced stretching. In the nucleation stage, it is assumed that the inherent defects at the grain boundaries are the nucleation sites of helium bubbles, and the number density of helium bubbles is proportional to the helium concentration. In the aggregation stage, the migration and coalescence of helium bubbles are mainly controlled by surface diffusion. As the temperature rises, the bubbles experience a growth process at high temperature, especially the absorption of helium/vacancies causes the size of the bubbles to increase. In the cooling stage, the growth of the bubbles is affected by the absorption of helium caused by the tensile stress, and the bubbles will expand further. This model can not only reflect the main physical mechanisms in the bubble evolution process but also reveal the detailed evolution laws of helium bubbles from nucleation to growth during the welding process by establishing precise mathematical equations.

By applying this model, it is possible to deeply explore the evolution behavior of grain boundary helium bubbles in austenitic stainless steel after neutron irradiation and reveal the influence of helium on the microstructure of the material. This provides a theoretical basis for understanding the propagation of cracks caused by helium and the mechanism of material performance degradation in a nuclear environment, especially helping to analyze the degradation of the mechanical properties of materials in high-temperature and irradiation environments. At the same time, this model provides important support for optimizing the selection and design of nuclear reactor materials, can effectively predict the performance of materials under different helium concentrations, temperatures, and stress conditions, and thus provides a theoretical guarantee for ensuring the long-term safe and stable operation of nuclear facilities.

## 2. Theoretical Model

The following assumptions are made for this model: (a) The evolution of helium bubbles at the grain boundaries during the welding process is divided into four characteristic stages: nucleation, aggregation, high-temperature growth, and cooling-induced stretching; (b) It is assumed that the bubbles nucleate uniformly at the grain boundaries; (c) The number density of nucleated bubbles is proportional to the helium concentration *C_He_*.

**Figure 3:**
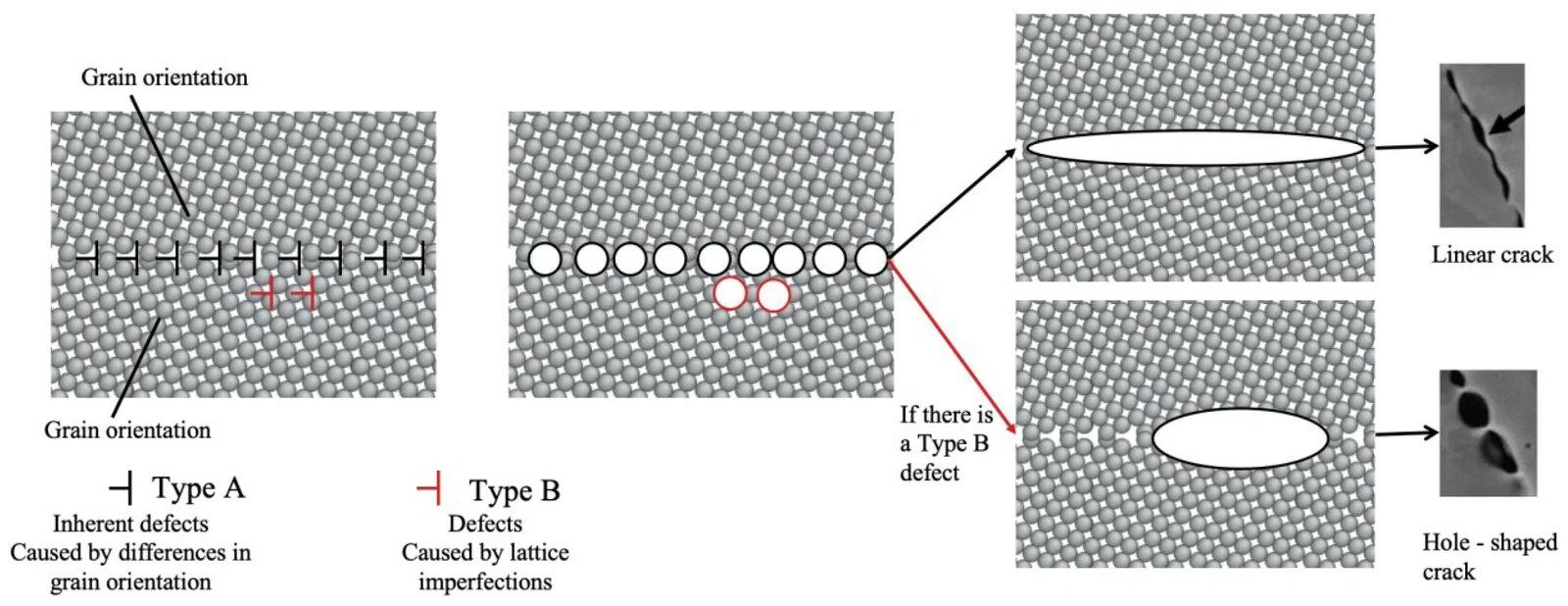
Schematic diagram of the mechanism of helium-induced cracks based on the crystal defect theory.

Based on the crystal defect theory and the measured size of helium bubbles, the development process of helium-induced cracks can be roughly divided into three stages according to their morphology: the formation of isolated bubbles or cavities, the interlinkage of grain-boundary cavities, and the propagation of localized cracks. It should be emphasized that these stages are classified by the dominant mechanisms and morphological features rather than by universal size thresholds. The theoretical model diagram of helium-induced cracks at the grain boundaries is shown in Figure 3. The experimental results of M. N. Gussev et al. clearly reveal the three stages in the development of helium-induced cracks: the helium bubble formation stage, the helium bubble aggregation stage, and the crack propagation stage. According to their experimental observations, the cracks in the heat-affected zone (HAZ) are intergranular, and the crack interiors are filled with voids generated by the growth of helium bubbles at grain boundaries. In the HAZ of the specimen containing 3 appm helium, helium bubbles together with a crack approximately 5 μm in length were observed; in the HAZ of the specimen containing 8 appm helium, at a distance of approximately 70 μm from the fusion boundary, several degraded and partially cracked grain boundaries were observed, with the helium bubbles exhibiting a characteristic morphological pattern and a tendency to aggregate into clusters [7]. In addition, the experiments conducted by C. A. Wang et al. [5] measured the average void-volume diameters on the fracture surfaces of the HAZ to be 0.96 μm and 0.92 μm for the steels containing 5.2 appm and 10 appm helium, respectively.

Although there have been many experimental and theoretical investigations of bubble nucleation under isothermal annealing conditions, there have been no reports of quantitative data for helium bubble nucleation under post-irradiation welding conditions. Based on the experimental phenomenon that the crack surface is filled with uniform and equal-sized (with a diameter of about 0.9 μm) voids caused by the growth of helium bubbles at the grain boundaries, for the four characteristic stages of the evolution of helium bubbles at the grain boundaries during the welding process—nucleation, aggregation, high-temperature growth, and cooling-induced stretching—a bubble nucleation model, a bubble migration and aggregation model, a bubble growth model during heating, and a bubble stretching model caused by tensile stress during cooling were established, respectively.

### 2.1. Bubble Nucleation Model

Before appreciable welding-induced growth, helium can be trapped at irradiation-induced defects and pre-existing grain-boundary defects, which can serve as sites for bubble nucleation. When the temperature rises but the fusion zone has not yet formed, the nucleated bubbles in the grain boundaries grow by absorbing helium, and at the same time, there is compressive stress hindering the growth of the bubbles. Theoretically, the molecular dynamics simulation technology should be used for the evolution of the behavior of helium bubbles at this stage. However, to simplify the model, it is assumed that the bubbles nucleate uniformly at the grain boundaries, and the surface number density *N_0_* of the nucleated bubbles is proportional to the helium concentration *C_He_* [11]:

*N*_0_ = *αC_He_* (1)

In Equation (1), N_0_ is the initial surface number density in m^−2^. C_He_ is entered as its numerical value in appm; for example, 3.2 appm is entered as 3.2, not 3.2 × 10^−6^. The adopted coefficient is α = 1.25 × 10^12^ m^−2^ appm^−1^ [11]. For a uniform two-dimensional population, the characteristic boundary area per bubble is L_0_^2^ = 1/N_0_, so

*L*_0_ = 1/√*N*_0_ = 1/√*(αC_He_);* *L*_0_
*[nm]* = 894.427/√*C_He_*. (2)

At C_He_ = 3.2 appm, N_0_ = 1.25 × 10^12^ × 3.2 = 4.0 × 10^12^ m^−2^ = 4.0 μm^−2^, giving L_0_ = 5.0 × 10^−7^ m = 500 nm. The same calculation gives 2000, 1000, 500, and 200 nm for 0.2, 0.8, 3.2, and 20 appm, respectively. If concentration is instead expressed as the atomic fraction x_He_ = 10^−6^ C_He_, the equivalent coefficient in N_0_ = α_x_ x_He_ is α_x_ = 1.25 × 10^18^ m^−2^. The mapping assumes unchanged effective trapping efficiency and grain-boundary structure; α is a model input, not a universal nucleation constant.

### 2.2. Bubble Migration and Aggregation Model

The migration process of bubbles is considered to be a random motion controlled by surface diffusion [12]. At this stage, the time change rate of the bubble density (N) is given by the following equation [13]:

(3) $$\frac{dN}{dt}=\frac{2\pi D_{b}N^{2}}{ln\left(L/4r\right)}$$

where L = 1/$\sqrt{N}$ is the bubble spacing, $D_{b}=D_{s}{\left(a/r\right)}^{4}$ is the bubble diffusion coefficient, $D_{s}$ is the bubble surface diffusion coefficient, and a is the lattice constant. It is assumed that the average radius r of the coalesced bubbles follows the law of volume conservation:

(4) $$r=r_{0}{\left(N_{0}/N\right)}^{1/3}$$

where $r_{0}$ and $N_{0}$ are the initial bubble radii and densities.

### 2.3. Bubble Growth Model During Heating

The growth of grain boundary bubbles during the heating process is mainly through the absorption of helium/vacancies (the stress in the fusion zone is 0). The growth rate of the bubbles is given by the following equation:

(5) $$\frac{\mathrm{dr}}{\mathrm{dt}}=\frac{\delta \Omega D_{gb}C_{v}^{e}}{2r^{2}}$$

(6) $$c_{v}^{e}(T)=exp(\frac{{\Delta S}_{v}}{k_{B}})\;exp(\frac{-{\Delta H}_{v}}{k_{B}T})$$

(7) $$C_{v}^{e}(T)=\frac{c_{v}^{e}(T)}{\Omega }$$

Here, $c_{v}^{e}(T)$ is the dimensionless equilibrium vacancy fraction per atomic site, and $C_{v}^{e}(T)$ is the vacancy number concentration in m^−3^ used in Equation (5). ${\Delta S}_{v}$ is vacancy formation entropy, ${\Delta H}_{v}$ is vacancy formation enthalpy, Ω is atomic volume, and $k_{B}$ denotes the Boltzmann constant previously written as k. The adopted diffusion input is D_gb_(T) = D_gb,0_ exp[−Q_gb_/(k_B_T)]. The parameters, units, and adopted sources are listed separately in Table 1.

Arrhenius expressions with activation energies in eV use k_B_ = 8.617333262 × 10^−5^ eV K^−1^. Equation (8), with stress in Pa and lengths in m, uses k_B_ = 1.380649 × 10^−23^ J K^−1^, so that σΩ and k_B_T are both energies.

The equilibrium approximation does not resolve irradiation-induced excess vacancies, trapping in helium–vacancy complexes, recombination with interstitials, or annihilation at sinks during welding. Retained excess vacancies may increase the supply, whereas trapping, recombination, and sink loss may reduce the mobile population. Without irradiation-specific initial conditions and coupled defect kinetics, the net error in the growth rate cannot be assigned a universal magnitude or sign. The equilibrium concentration is therefore a reference kinetic assumption rather than a measured post-irradiation vacancy population.

According to the literature, it is assumed that the bubble growth model during the heating period is applicable at temperatures above approximately 900 K [10]. The prescribed transition temperature is an additional model assumption. Its influence on the calculated growth history requires a separate sensitivity assessment.

Notably, in the temperature range of 600–900 K, helium migration is dominated by transient diffusion behavior. Although such diffusion cannot induce immediate bubble nucleation, it promotes the long-term aggregation of helium atoms at grain boundaries. This low-temperature transient diffusion process does not directly generate helium bubbles, but it provides a prerequisite for the rapid and pronounced bubble growth that occurs once the temperature exceeds 900 K.

### 2.4. Bubble Stretching Model Caused by Tensile Stress During Cooling

In the cooling stage, the growth of bubbles is based on the absorption of helium caused by tensile stress. The growth rate of grain boundary bubbles under tensile stress is given by the following equation [14]:

(8) $$\frac{\mathrm{dr}}{\mathrm{dt}}=\frac{2\pi \delta \Omega D_{gb}\sigma }{LrkT}$$

where σ is the tensile stress perpendicular to the grain boundary, and k is the Boltzmann constant. The tensile stress (σ)is simulated using elastic theory [12]:

Σ = Eα_l_[T_max_ − T] (9)

where *E* is the Young’s modulus, *α_l_* is the coefficient of thermal expansion, and *T_max_* is the peak temperature during the welding process. The unit for temperature is Kelvin (K). The value of 200 MPa is an arbitrary, analyst-selected cap for the parametric analysis; it is not assigned to the high-temperature yield strength of a specified steel. During cooling, the capped history is σ(t; σcap) = min{σel(t), σcap}, where σel(t) = max{E[T(t)]αl[Tmax − T(t)], 0} and σcap = 200 MPa. This truncation does not resolve plasticity, creep, or the actual weld stress history. Its sensitivity is assessed analytically in Section 4.3.

## 3. Research Method

**Figure 4:**
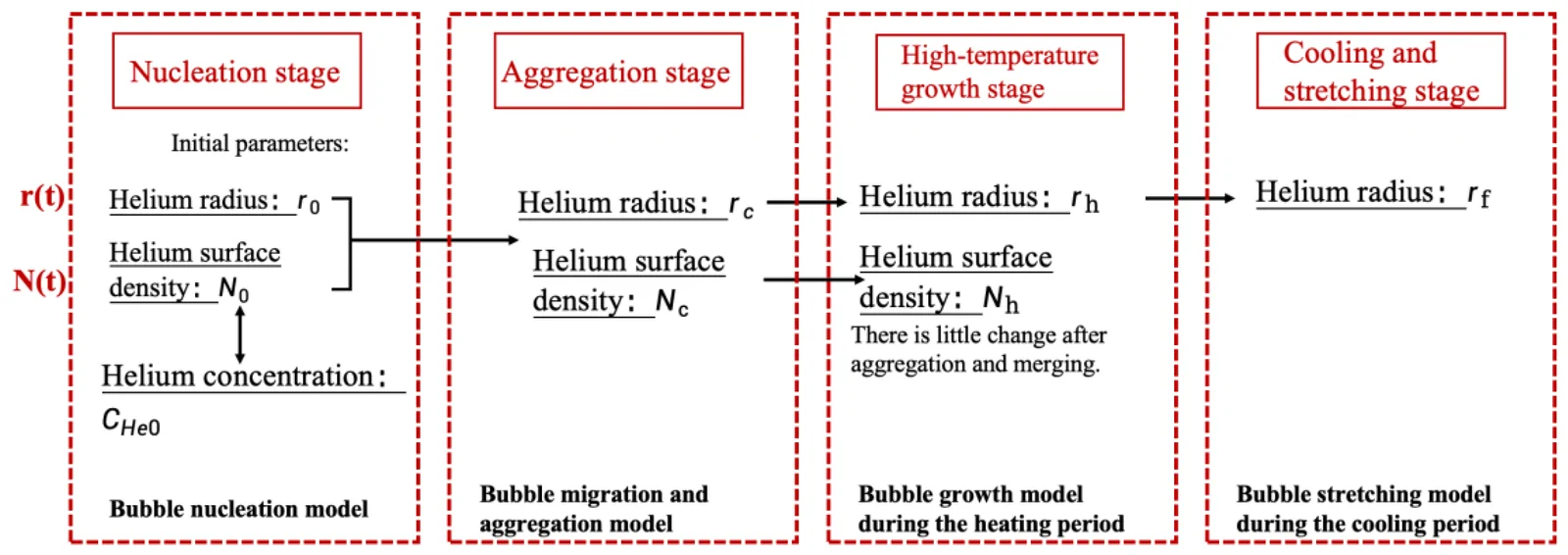
Technical roadmap of the model for the nucleation, coalescence, and growth of helium bubbles at the grain boundaries.

Figure 4 illustrates the specific technical route of this theoretical model. Corresponding equations are used in different stages to calculate the evolution laws of the radius and density of helium bubbles over time so as to obtain the kinetic parameters of helium bubble growth, such as the influence laws of helium concentration, temperature, and stress on the growth of helium bubbles. Furthermore, the evolution laws of the density and diameter of helium bubbles over time under different welding heat input conditions are obtained. In the figure, r(t) is the time-dependent helium bubble radius, and N(t) is the time-dependent helium surface density.

## 4. Analysis of Kinetic Parameters of Helium Bubble Growth

In this model, gas tungsten arc welding (GTAW) is adopted for single-pass surfacing tests. The bubble diameter within the heat-affected zone (HAZ) under different heat input conditions is calculated. The temperature–time relationship during welding is derived based on the heat conduction analysis of a moving point heat source in an infinite solid. Iterative calculations of Equations (3)–(8) are performed to obtain the bubble diameter and bubble density. The parameters used in the calculation are listed in Table 1. The two values of kB represent the same constant in different energy units.

**Figure 5:**
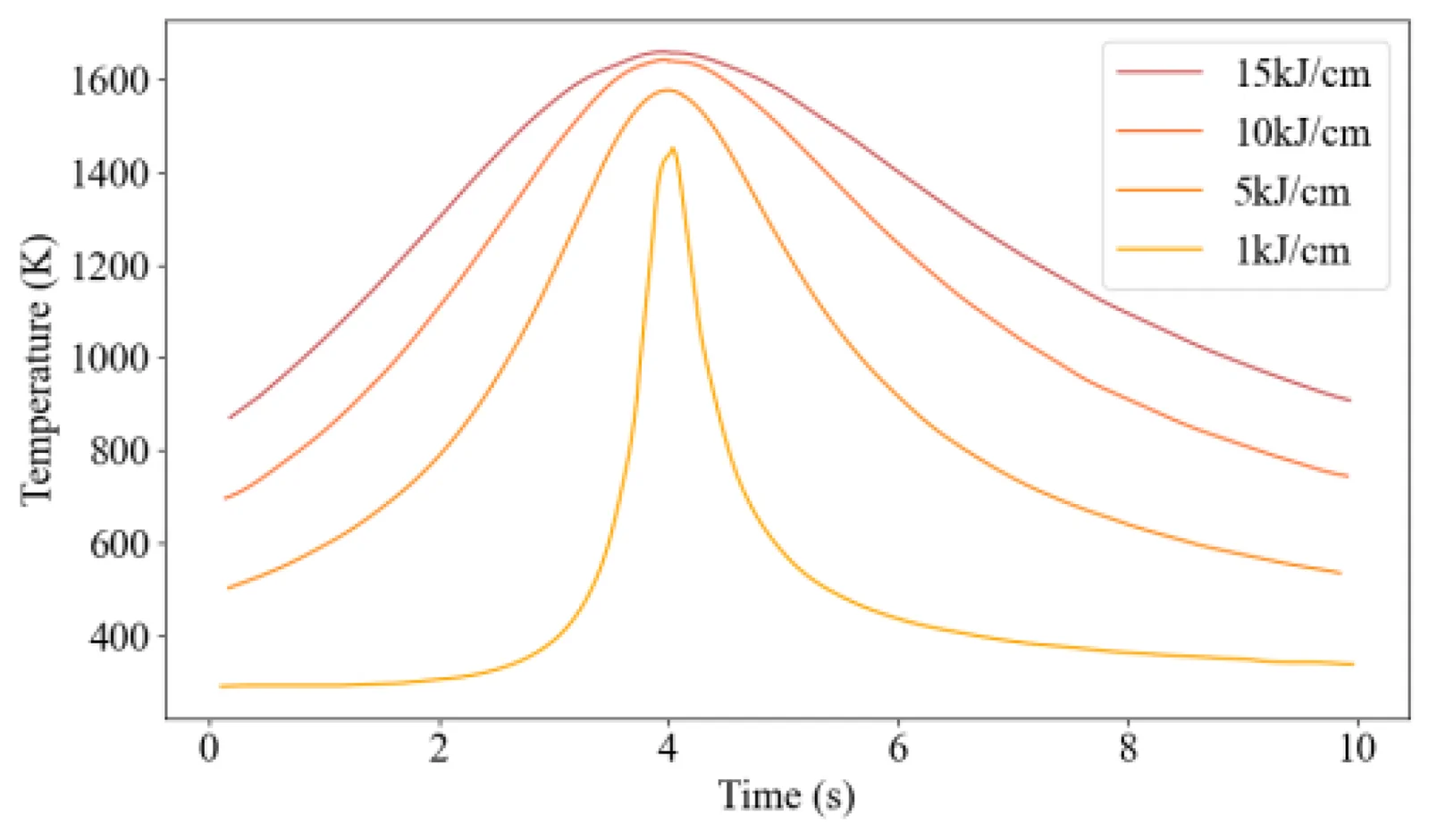
Curves of the temperature of the welding thermal cycle changing with time under different welding heat input conditions.

Figure 5 presents the welding thermal cycle temperature curves of the heat-affected zone at a position 0.1 mm from the weld fusion line under different welding heat input conditions. The calculated results of the present model are in good agreement with the research results of Kawano et al. [11].

In this study, the evolution law of the diameter of helium bubbles over time and the final diameter of helium bubbles after welding are used as the characterization quantities to analyze the influence of different helium concentrations, temperatures, and stresses on the growth kinetics of helium bubbles.

Generally speaking, the range of the initial helium atom concentration *C_He_* of austenitic stainless steel in a nuclear reactor is 0 to 100 appm. In the model, the initial bubble spacing *L_0_* is taken as a parameter. When *L*_0_ is 2000 nm, 1000 nm, 500 nm, and 200 nm, the corresponding initial helium concentrations are 0.2 appm, 0.8 appm, 3.2 appm, and 20.0 appm; the stress σ ranges from 0 to 200 MPa, and the specific stress values are 50 MPa, 100 MPa, 150 MPa, and 200 MPa.

### 4.1. Influence of Helium Concentration on the Growth of Helium Bubbles

To study the influence of helium concentration on the growth of helium bubbles, the initial bubble spacing, that is, the helium concentration, is controlled as the only variable. At this time, the welding heat input in the model is 15 kJ/cm, and the stress is 200 MPa. Taking the diameter of helium bubbles as the characterization quantity, the evolution law of the diameter of helium bubbles over time is studied.

**Figure 6:**
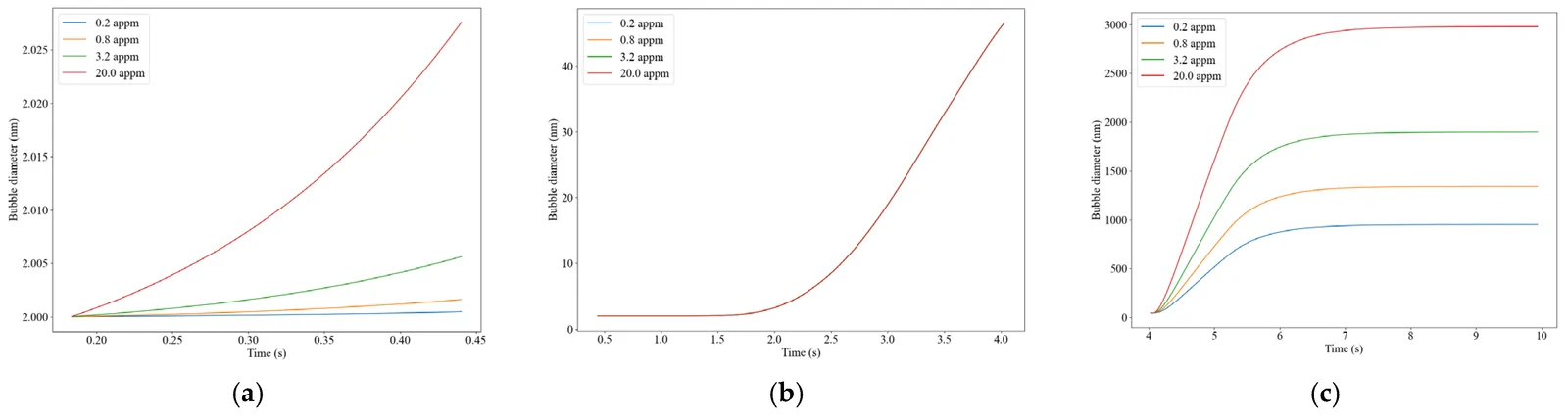
Evolution law of helium bubble diameter with time under different initial helium concentrations: (**a**) diagram of the change in bubble diameter with time during the aggregation stage; (**b**) diagram of the change in bubble diameter with time during the heating stage (four lines overlap); (**c**) diagram of the change in bubble diameter with time during the cooling stage.

The results in Figure 6 show that during the aggregation stage, the helium concentration has little influence on the diameter of helium bubbles, and the difference in the diameters of the bubbles with 0.2 appm and 20.0 appm is only 2%. During the heating stage, the helium concentration has no significant influence on the evolution of the bubble diameter, indicating that the diameter of helium bubbles at this stage is mainly affected by the mechanisms of thermal expansion and thermal vacancy absorption, and the interaction between a single helium bubble and the surrounding helium bubbles can be neglected. During the cooling stage, as the helium concentration increases, the diameter of the helium bubbles after welding increases, showing a significant non-linear relationship. When the helium concentration increases from 0.2 appm to 20.0 appm, the diameter of the helium bubbles after welding only increases by about 3 times.

### 4.2. Influence of Temperature on the Growth of Helium Bubbles

**Figure 7:**
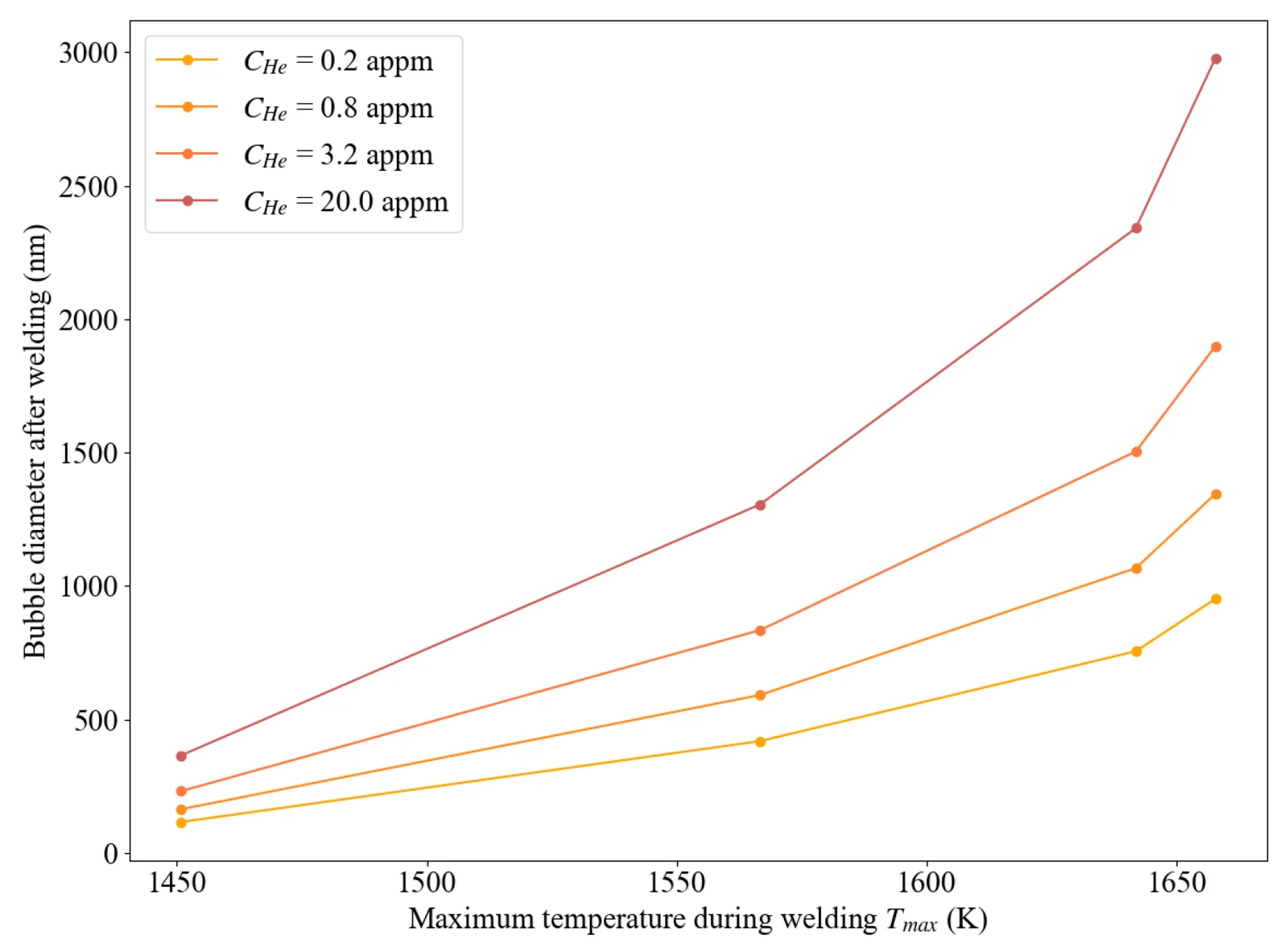
Curve of the relationship between the peak temperature in the heat-affected zone and the helium bubble diameter after welding under different helium concentration conditions.

Figure 7 presents the relationship curves between the peak temperature of the heat-affected zone (HAZ) (1450 K, 1566 K, 1641 K, and 1657 K) and the post-weld helium bubble diameter under different helium concentrations at various welding heat inputs. The results indicate that the HAZ peak temperature has a significant non-linear effect on the final helium bubble diameter. At lower peak temperatures, the helium bubbles are generally smaller. As the peak temperature increases, the coalescence and growth of helium bubbles accelerate, leading to a significant increase in the post-weld diameter. This growth trend becomes particularly pronounced when the temperature exceeds 1641 K, exhibiting a characteristic critical temperature. Shi-Hao Li et al. [3]. revealed a similar conclusion: the diffusivity of helium atoms increases significantly at high temperatures (especially during the annealing process at around 600 °C), and the enhanced mobility promotes the aggregation of helium atoms into larger bubbles, thereby leading to more obvious material embrittlement and structural failure.

Furthermore, the effect of peak temperature is strongly coupled with helium concentration. At the same peak temperature, a higher initial helium concentration leads to a larger final bubble diameter. In particular, as the temperature approaches 1657 K, the diameter growth curves become much steeper for the higher concentration cases (e.g., 3.2 appm and 20.0 appm). This indicates that a higher peak temperature promotes thermal vacancy absorption and thermal expansion mechanisms, providing a stronger driving force for bubble growth; under the synergistic effect of high temperature and high concentration, the coarsening and aggregation of helium bubbles are drastically enhanced.

### 4.3. Influence of Stress on the Growth of Helium Bubbles

**Figure 8:**
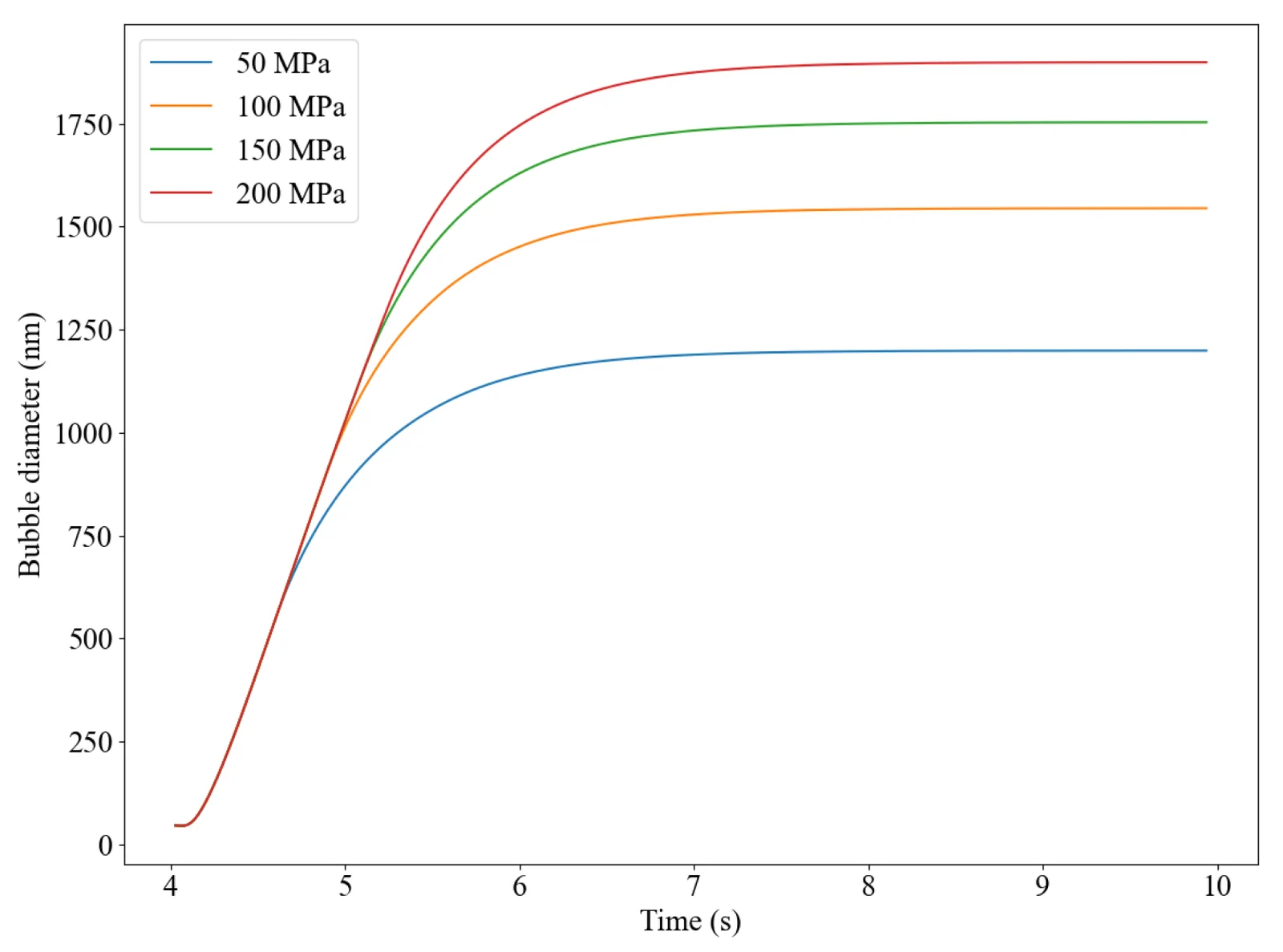
Evolution law of helium bubble diameter with time under different tensile stresses during the cooling stage.

**Figure 9:**
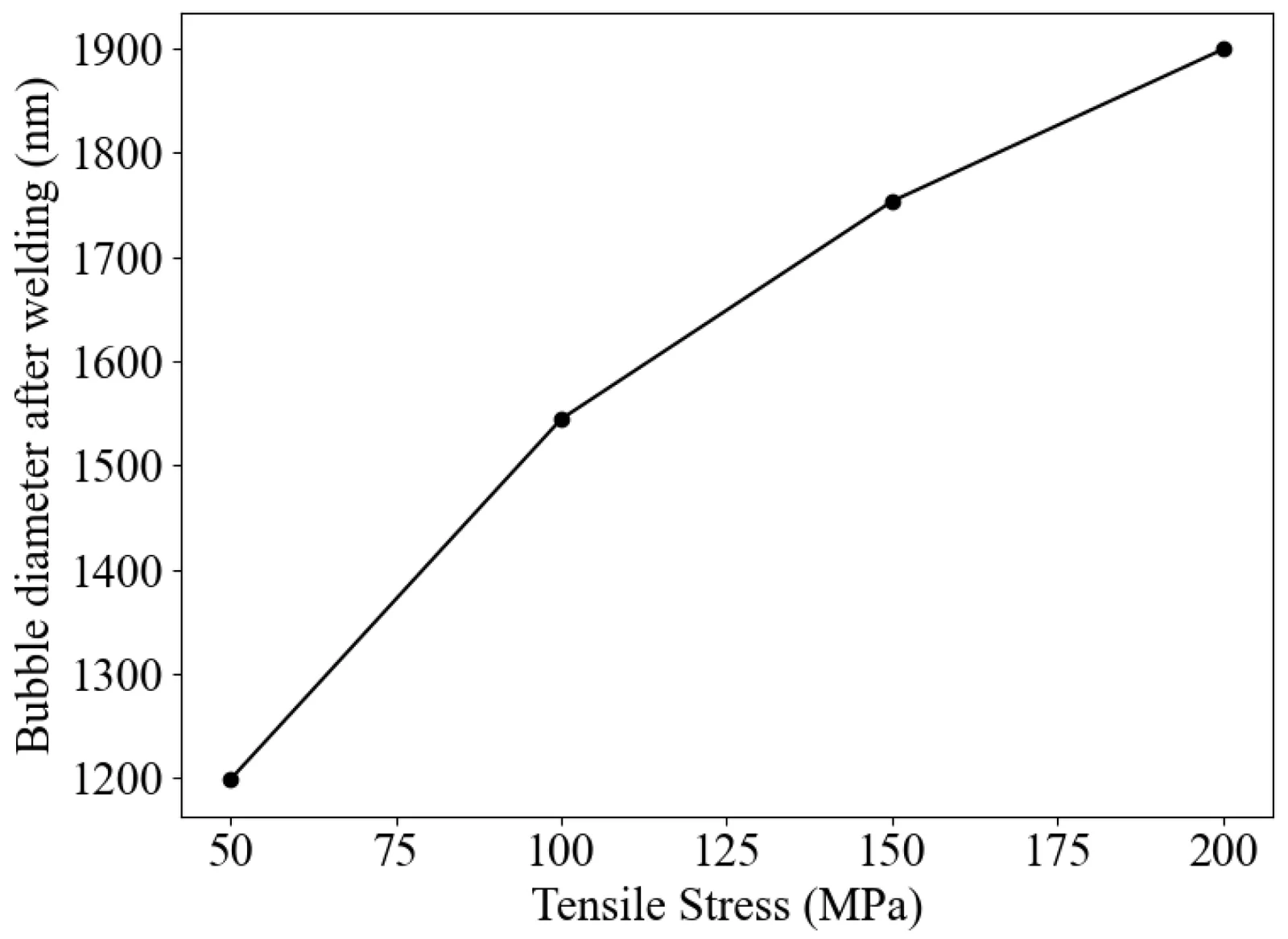
Relationship between the maximum diameter of helium bubbles after welding and the stress.

When studying the influence of stress on the growth of helium bubbles, the stress is controlled as the only variable, the initial bubble spacing is 500 nm, and the heat input is 15 kJ/cm. Since the material is mainly affected by tensile stress during the cooling stage of welding, the model only considers the change trend of the diameter of helium bubbles under different tensile stresses during the cooling stage of welding. The corresponding evolution laws of the diameter of helium bubbles are shown in Figure 8 and Figure 9. The results shown in Figure 8 indicate that under the action of tensile stress, the growth of helium bubbles is accelerated. The greater the tensile stress, the larger the diameter of the helium bubbles after welding. It should be noted that the results at 50, 100, 150, and 200 MPa are parametric analyses under prescribed stress conditions (see Section 2.4), rather than predictions of the actual stress history in a specific weld.

The results shown in Figure 9 indicate that the greater the tensile stress, the larger the diameter of the helium bubbles after welding. From 50 to 200 MPa tensile stress, as the tensile stress increases by the same amount, the growth amplitude of the diameter of the helium bubbles after welding gradually decreases. Similarly, the research of Braski et al. shows that at the intersections of double-grain boundaries and triple-grain boundaries, the sliding caused by stress promotes the coalescence and growth of bubbles. Compared with the stress-free condition, the tensile stress increases the growth rate of bubbles in these areas [15,16,17,18].

For the capped history defined in Section 2.4, let A(t) = 2πδΩD_gb_[T(t)]/{L(t)k_B_T(t)} ≥ 0. With rh denoting the radius at the start of cooling and r_f_ the final radius, Equation (8) integrates over the cooling stage as

(10) $$r_{f}^{2}-r_{h}^{2}=2\int A(t)\sigma (t;\sigma_{cap})dt$$

To compare caps, the temperature history, spacing history, and r_h_ are held fixed. At every time, 0.75σ(t; 200 MPa) ≤ σ(t; 150 MPa) ≤ σ(t; 200 MPa), and σ(t; 200 MPa) ≤ σ(t; 250 MPa) ≤ 1.25σ(t; 200 MPa). Applying the same nonnegative weighting A(t) and writing d_f_ = 2r_f_ gives

(11) $$\surd 0.75\;\leq \frac{d_{f}f(150)}{d_{f}(200)}\leq \;1;\;1\;\leq \frac{d_{f}(250)}{d_{f}(200)}\leq \;\surd 1.25$$

Reducing the cap from 200 to 150 MPa can therefore decrease the nominal final diameter by at most 13.4%, whereas raising it to 250 MPa can increase the diameter by at most 11.8%, under these assumptions. The change is smaller if the cap is inactive for part of the growth period or the initial radius r_h_ is appreciable. These are bounds of the adopted growth law, not additional welding-cycle simulations. They exclude feedback from plasticity, creep, and cavity interactions.

This comparison concerns the explicitly capped history. It does not reconstruct the loading used in Figure 8 and Figure 9. For a constant prescribed stress, Equation (10) makes r_f_^2^ − r_h_^2^ proportional to that stress only when the other histories are identical. Diminishing diameter increments do not establish physical saturation at 200 MPa.

The above results provide a theoretical basis for the helium-induced crack suppression technology based on stress improvement. Wang et al. found that applying compressive stress during the welding process can suppress the helium-induced cracks that occur when there is no additional stress control [5]. The auxiliary beam stress-improved laser welding technology developed by the Oak Ridge National Laboratory (ORNL) in the United States and the Electric Power Research Institute (EPRI) in the United States can actively improve the stress during the laser repair welding of high-irradiated reactor internal components to avoid helium-induced cracks [19].

## 5. Analysis of the Evolution Laws of Helium Bubble Density and Diameter

Furthermore, based on the model of the nucleation, migration, aggregation, and growth of helium bubbles in the heat-affected zone during the welding process, the evolution laws of the density and diameter of helium bubbles over time under different welding heat input conditions were studied. In the model, the initial helium concentration was set to 3.2 appm, that is, the initial bubble density was 4 bubbles/μm^2^, and the initial stress was set to 200 MPa. The evolution laws of the density and diameter of helium bubbles over time under different welding heat input conditions are as follows.

### 5.1. Evolution Law of Helium Bubble Density

**Figure 10:**
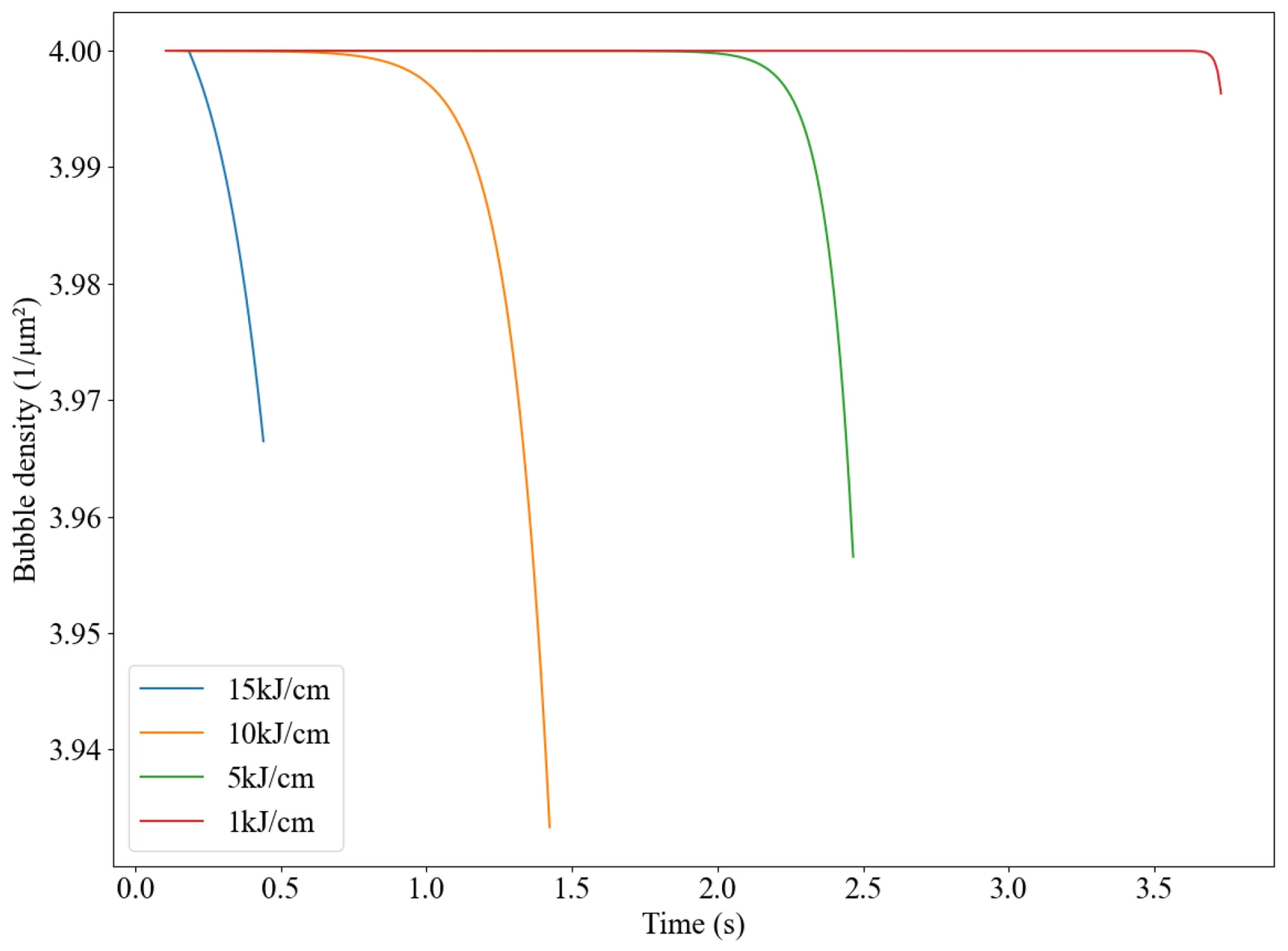
Relationship between the density of helium bubbles and time during the aggregation stage.

The change in the number of grain-boundary helium bubbles mainly occurs during the aggregation stage. The random movement controlled by surface diffusion within the grain boundaries leads to the coalescence of helium bubbles, and its quantitative relationship is shown in Figure 10. As can be seen from Figure 10, the final value of the helium-bubble density first decreases and then increases with the increase in the heat input. Within a certain range, the greater the heat input, the higher the peak temperature in the heat-affected zone, the greater the driving force for the migration and aggregation of helium bubbles, and the faster the decrease in the helium-bubble density. This is because the surface diffusion of helium bubbles within the grain boundaries is promoted by the increase in temperature. When the heat input is too large, the evolution process of helium bubbles will quickly change from the aggregation stage to the high-temperature growth stage. The grain-boundary bubbles will migrate and aggregate rapidly. However, due to the excessive heat input, the peak temperature will quickly exceed 923 K (the switching temperature to the high-temperature growth calculation stage [10]). At this time, the growth mechanism of grain-boundary helium bubbles, which is the absorption of helium/vacancies in the grain interior, exceeds the surface-diffusion mechanism within the grain boundaries, and thus the number of grain-boundary helium bubbles no longer decreases further. Therefore, under higher heat input, both the bubble density and the bubble diameter are at a relatively high level, making it easier to generate helium-induced cracks.

### 5.2. Evolution Law of Helium Bubble Diameter

With the progress of the welding process, the nucleated helium bubbles will further expand by absorbing surrounding helium atoms and vacancies, and the diameter of the helium bubbles will continue to increase.

**Figure 11:**
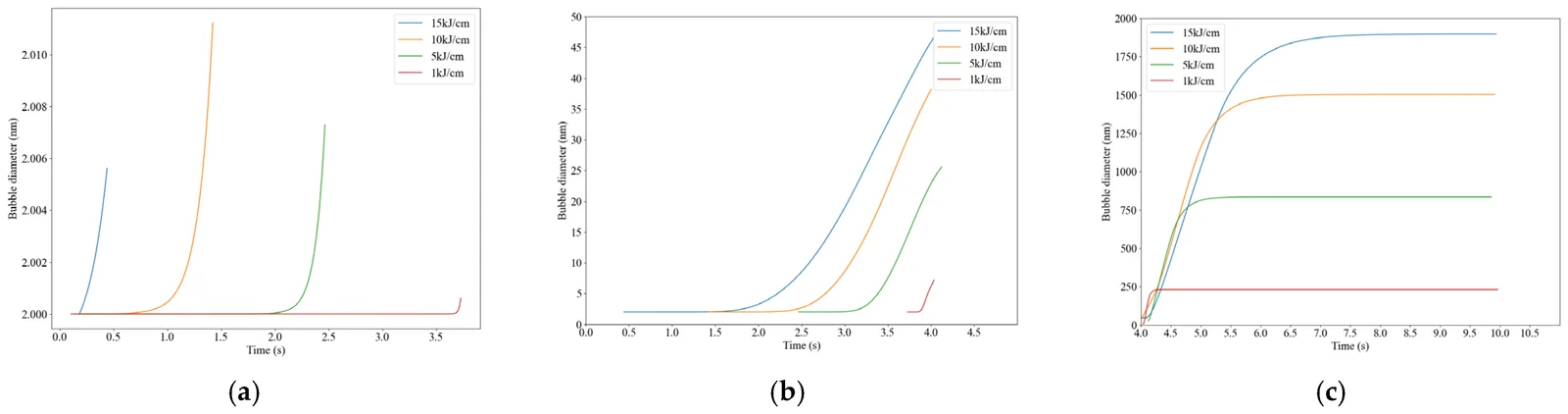
Diagrams of the change of helium bubble diameter with time in different stages: (**a**) diagram of the change in bubble diameter with time during the aggregation stage; (**b**) diagram of the change in bubble diameter with time during the heating-up stage; (**c**) diagram of the change in bubble diameter with time during the cooling-down stage.

As shown in Figure 11, the evolution of the diameter of helium bubbles shows stage characteristics. During the aggregation stage, the diameter increases by only within 0.01 nm. During the heating stage, the diameter increases to 10–50 nm. During the cooling stage, the diameter increases to 100–2000 nm. During the aggregation and heating stages, the diameter of the helium bubbles shows a monotonically increasing trend. During the cooling stage, after experiencing a large increase, the diameter of the helium bubbles will approach a stable value and no longer continue to increase.

The greater the heat input, the faster the transition of helium bubbles from the aggregation stage to the high-temperature growth stage occurs, and the greater the increase in the diameter of helium bubbles during the heating stage. The greater the heat input, the longer the growth time of helium bubbles during the cooling stage, and the larger the final size of the helium bubbles, showing a non-linear relationship.

## 6. Conclusions

This study focuses on the evolution of helium bubbles in the heat-affected zone of irradiated stainless steel. Regarding the nucleation, aggregation, and growth model of helium bubbles at the grain boundaries, a numerical model covering four characteristic stages of nucleation, aggregation, high-temperature growth, and cooling-induced stretching was established based on the numerical model of uniform distribution of helium bubbles. The possible mechanism of the formation of helium-induced cracks caused by the migration and aggregation of helium bubbles in the welding heat-affected zone was clarified. By simulating the evolution of helium bubbles under different helium concentrations, temperature fields, and stress fields, the influence of kinetic parameters of helium-bubble growth on the number, size, and growth rate of helium bubbles was discussed, and the action mechanisms of key parameters such as helium concentration, stress level, and welding heat input on the growth of helium bubbles were revealed:

(1) Helium concentration has little effect on helium bubble growth during the aggregation and temperature-rise stages. In the cooling stage, the helium bubble diameter increases nonlinearly with the rise of helium concentration. When the helium concentration increases from 0.2 ppm to 20.0 ppm, the post-weld helium bubble diameter increases by approximately three times.

(2) As the temperature increases, the bubbles coalesce and grow faster, and the diameter of the helium bubbles after welding is larger.

(3) Under tensile stress, the growth of helium bubbles is accelerated. The greater the tensile stress, the larger the diameter of the helium bubbles after welding.

(4) The analysis of the nucleation, coalescence, and growth mechanisms of helium bubbles further explores the quantitative relationship of the evolution laws of the number and diameter of helium bubbles under different welding heat input conditions. The results show obvious stage characteristics, revealing the promoting effect of high temperature on helium-induced cracks.

## Figures and Tables

**Table 1:** Parameter values used in calculation [11].

Parameter	Value or Expression	Unit
Vacancy formation entropy ΔS_v_	1.5 k_B_	J K^−1^ or eV K^−1^
Vacancy formation energy ΔH_v_	1.8	eV
Atomic volume Ω	1.0 × 10^−29^	m^3^
Boltzmann’s constant k_B_	1.380649 × 10^−23^	J K^−1^
Boltzmann’s constant k_B_	8.617333262 × 10^−5^	eV K^−1^
Self-diffusion coefficient in the GB D_gb,0_	2.0 × 10^−4^	m^2^ s^−1^
Q_gb_	1.65	eV
Self-diffusion coefficient in the GB D_gb_(T)	D_gb,0_ exp[−Q_gb_/(k_B_T)]	m^2^ s^−1^
the dimensionless equilibrium vacancy fraction per atomic site c^ᵉ^_v_(T)	Equation (6)	dimensionless
vacancy number concentration C^ᵉ^_v_(T)	Equation (7)	m^−3^
Initial bubble radius *r*_0_	1	nm
Grain boundary thickness *δ*	0.4	nm
Lattice spacing a	0.2	nm
Surface diffusion coefficient *D_s_*	0.4·exp(−2.2 eV/kT)	m^2^/s
Work hardening rate *n*	0.3	/
Thermal expansion coefficient *α_l_*	2×10^−5^	K^−1^
Young’s modulus *E*	[243.9−0.1078 T] × 10^9^	N/m^2^
Initial bubble spacing *L*_0_	50–2000	nm
Weld heat input *Q*	1–15	kJ/cm

## Data Availability

The original contributions presented in this study are included in the article. Further inquiries can be directed to the corresponding author.
